# Development of a Minimum Dataset for the Global Monitoring of the Safety and Efficacy of Growth Hormone Replacement in Adults With Growth Hormone Deficiency (AGHD)

**DOI:** 10.1111/cen.15298

**Published:** 2025-07-21

**Authors:** Suet Ching Chen, Angela K. Lucas‐Herald, Ruoning Tang, Xanthippi Tseretopoulou, Malika Alimussina, Deno Andrews, Beverly M. K. Biller, Cesar L. Boguszewski, Jillian Bryce, Minglu Chen, Peter E. Clayton, Maria Fleseriu, Judith Gebauer, Ken K. Y. Ho, Jens Otto L. Jorgensen, Xiaoping Luo, Bradley S. Miller, Sebastian JCMM. Neggers, Lars Sävendahl, Katharina Schilbach, Christian J. Strasburger, Yutaka Takahashi, Diana Vitali, Kevin C. J. Yuen, Andrew R. Hoffman, Gudmundur Johannson, S. Faisal Ahmed

**Affiliations:** ^1^ Developmental Endocrinology Research Group, Royal Hospital for Children University of Glasgow Glasgow UK; ^2^ Office for Rare Conditions University of Glasgow Glasgow UK; ^3^ Chicago Illinois USA; ^4^ Neuroendocrine & Pituitary Tumor Clinical Center Massachusetts General Hospital Boston Massachusetts USA; ^5^ Endocrine Division (SEMPR), Department of Internal Medicine Federal University of Parana Curitiba Brazil; ^6^ University of Manchester Manchester UK; ^7^ Departments of Medicine and Neurological Surgery, Pituitary Center Oregon Health & Science University Portland Oregon USA; ^8^ Department of Internal Medicine 1 University Hospital of Schleswig‐Holstein Luebeck Germany; ^9^ The Garvan Institute of Medical Research and St. Vincent's Hospital Sydney New South Wales Australia; ^10^ Aarhus University Hospital Aarhus Denmark; ^11^ Tongji Hospital Huazhong University of Science and Technology Wuhan China; ^12^ MHealth Fairview Masonic Children's Hospital University of Minnesota Medical School Minneapolis Minnesota USA; ^13^ Department of Internal Medicine Section Endocrinology and Pituitary Center Rotterdam The Netherlands; ^14^ Department of Women's and Children's Health Karolinska Institutet and Karolinska University Hospital Stockholm Sweden; ^15^ Department of Medicine IV LMU University Hospital, LMU, Munich Munich Germany; ^16^ Deggendorf Institute of Technology Deggendorf Germany; ^17^ Department of Endocrinology and Metabolic Disorders Charite Universitaetsmedizin Berlin Germany; ^18^ Department of Diabetes and Endocrinology Nara Medical University Kashihara Japan; ^19^ SOD ITALY APS Italian Patients Organisation for Septic Optic Dysplasia and Other Neuroendocrine Diseases Rome Italy; ^20^ Barrow Pituitary Center University Arizona College of Medicine and Creighton University School of Medicine Phoenix Arizona USA; ^21^ Department of Medicine Stanford University School of Medicine Stanford California USA; ^22^ Sahlgrenska University Hospital and Institute of Medicine, Sahlgrenska Academy University of Gothenburg Gothenburg Sweden

**Keywords:** adults, core outcomes, effectiveness, growth hormone, growth hormone deficiency, growth hormone replacement, safety

## Abstract

**Objective:**

To identify the minimum dataset (MDS) for the monitoring of safety and effectiveness of GH in adults with growth hormone deficiency (AGHD).

**Design:**

Systematic review and expert consensus.

**Methods:**

Outcomes for AGHD were identified through a systematic literature search in PubMed, Science Direct and Cochrane. In addition, 17 clinical experts from 10 countries and two patient representatives assembled through the Global Registry for Novel Therapies in Rare Bone and Endocrine Conditions (GloBE‐Reg) provided data items that ideally should be collected routinely. These items were subsequently graded independently by participants on: (1) importance of the data field and (2) ease of data collection in routine clinical practice.

**Results:**

The systematic review identified 35 studies with 6732 participants with AGHD with a median age of 49 (range, 22–82) years. The common outcome categories included were cardiovascular in 21 (60%) studies, serum IGF‐I in 13 (37%) and IGF‐I SDS in 8 (23%), adiposity measures in 15 (44%) and psychosocial outcomes in 10 (29%). A total of 190 items were provided by experts and 86 (45%) achieved sufficient consensus and alignment with reported outcomes to create a final MDS with 45 items to be assessed, of which only 43 are manually entered.

**Conclusions:**

This study has identified by consensus a minimum dataset considered necessary to provide consistency and comparability in global studies of AGHD.

## 1 Introduction

Recombinant human growth hormone therapy (GH) has been available since the mid‐1980s for paediatric growth hormone deficiency (GHD) where it clearly reverses impaired growth rate [1]. Since the mid‐1990s GH has also been available and approved for adult GHD (AGHD) where it may exert a wide range of beneficial effects on body composition, exercise capacity, cardiovascular function, skeletal integrity, cognition and quality of life [2, 3, 4, 5] in patients with isolated GHD and in patients with panhypopituitarism [6]. Although GH therapy for AGHD has been recommended in clinical practice guidelines from the Endocrine Society, the Growth Hormone Research Society and the American Association of Clinical Endocrinologists [3, 7, 8, 9], several unanswered questions remain, including adherence, quality of life, safety and effectiveness. These points have become essential to address with the recent introduction and availability in some countries of long‐acting formulations of GHs (LAGH) [10]. However, longer term safety and efficacy of GH in AGHD are difficult to interpret by the lack of consistency in data collection among studies. Given that these questions require collection of clinical data over many years in AGHD patients, there is a need to identify the core outcomes to be reported in a standardised manner in all therapeutic intervention trials [11].

These core outcomes require a minimum dataset (MDS) that can be sustainably and consistently collected in a routine clinical setting over the lifespan of an individual who is receiving GH. Not only would this allow the reporting of a core set of outcomes as a minimum, as it would also standardise the reports, reducing variability and improving the quality of data collection whilst minimising the burden of data entry by health care providers [12]. The Global Registry for Novel Therapies in Rare Bone and Endocrine Conditions (https://globe-reg.net), a new initiative aimed at gathering real‐world data on safety and effectiveness of novel therapies in endocrinology, has recently developed a MDS for monitoring safety and effectiveness of GH for childhood GHD [11]. The aim of the current study was to perform a systematic review of the core outcome measures that are commonly reported in the field of AGHD and combine this with the same methodology as described recently for childhood GHD [11] to develop a MDS that could be collected in the routine clinical setting for AGHD.

## 2 Methods

### 2.1 Search Strategy of Systematic Review

Systematic computerised literature searches were performed in PubMed, Science Direct (previously Embase), and Cochrane in June 2023. This literature search was based on the following PICO (Population, Intervention, Comparison, Outcome) question:

- P: patients started on GH for any form of GHD and age at starting equal to or over the age of 16 years
- I: GH for GHD
- C: any
- O: any safety and effectiveness outcomes

Studies that met the eligibility criteria were included with no language or date limitations. The Medline search algorithm was: (‘Growth Hormone’ [Mesh] OR ‘Somatotropin’) AND (‘outcome’ OR ‘effectiveness’ OR ‘efficacy’ OR ‘safety’) AND (‘adult’ NOT ‘child’ OR ‘children’). The references of the latest systematic reviews and meta‐analyses on the topic were also checked, and any eligible studies were included.

### 2.2 Evaluation of Studies Included in Systematic Review

The systematic review was performed in accordance with the Preferred Reporting Items for Systematic Reviews and Meta‐analysis guidelines (PRISMA) [13]. Two members of the research team (RT and ALH) independently reviewed the titles, abstracts, and full texts of all studies identified by the search in a sequential fashion, to identify which were eligible for inclusion using the Covidence systematic review software, Veritas Health Innovation, Melbourne, Australia (www.covidence.org). Any discrepancies were resolved by discussion with a third member of the team (XT). The reasons for excluding abstracts and full texts were recorded. Neither of the review authors were blind to the journal titles or to the study authors or institutions. Studies were excluded if they were not about humans or patients aged over 16 or did not use GH for treatment for GHD. Case reports were excluded if they did not have more than two patients. Studies were also excluded if the only outcome reported was treatment adherence, as this was not considered to be an outcome of GH therapy. For each of the included studies, the epidemiological design, the journal title, authors, year of publication, country of origin, study sample, population age, the outcome(s) assessed, the tool which was used to measure the outcome, and the frequency of the measurement were recorded. Assessment of bias was undertaken using the Cochrane tool, available on the Covidence platform. This was adapted to observational studies using the ROBINS‐E checklist [14]. This was again performed independently by two reviewers (RT, ALH) and disagreements were resolved by discussion with a third reviewer (XT).

### 2.3 Development of the Minimum Dataset (MDS)

Seventeen international clinical experts from 10 countries and the pharmaceutical industry and two representatives from patient organisations, who were members of the Adulthood GHD Expert Working Group (EWG) in GloBE‐Reg, participated in the development of the MDS using the methodology described previously [8]. A comprehensive list of data items collected by the clinicians on routine clinical visits in their centres and those from the systematic review of core outcomes conducted were compiled. Each participant was asked to determine the: (i) importance of the data field and (ii) ease of data collection as part of routine clinical practice, using a traffic light grading system. The importance of the data field referred to the level of clinical care for establishing the diagnosis, assessing efficacy, and monitoring for adverse events. The ease of data collection was based on the participants’ experience on how readily available the information was in routine clinical settings. Data fields that achieved 70% or greater consensus in terms of importance qualified for the MDS, provided that fewer than 50% of the respondents deemed the item difficult to collect. The development of the final MDS occurred over 4 months in 2024, which included two virtual meetings among the EWG members. The initial meeting was used to clarify the process, and this was followed by independent scoring of the data items by the participants, while the second meeting was used to discuss the results before the final MDS was developed.

## 3 Results

### 3.1 Systematic Review Study Selection

**FIGURE 1:**
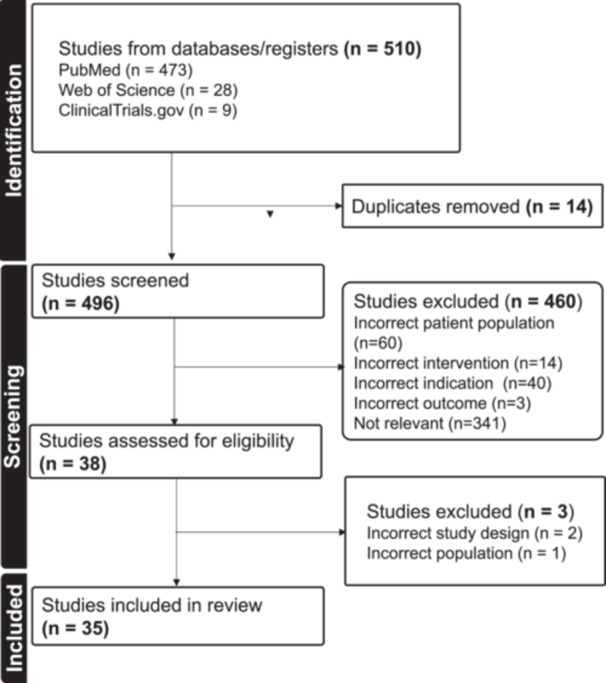
Study selection strategy flowchart in accordance with PRISMA guidelines.

Using the search strategy, a total of 510 articles were initially screened, with 473 (93%) from PubMed, 28 (5%) from Web of Science, and 9 (2%) from ClinicTrials.gov. After excluding 14 duplicates, 496 (97%) articles were screened by titles and abstracts. Of these, 60 (12%) were excluded because they were only about patients < 16 years of age; 40 (8%) because they were not related to GHD but to other indications for GH therapy; 14 (3%) because they did not consider GH treatment; 3 (1%) articles because they focused on adherence; and 341 (69%) articles because they were not relevant to GH or AGHD. A full‐text evaluation was conducted on the remaining 38 articles, and another three articles were excluded for the following reasons: two due to incorrect study design (being systematic reviews); and one due to wrong patient population. After selection, 35 articles were eligible for inclusion in the study (Figure 1).

### 3.2 Characteristics of Included Studies

The characteristics of the 35 eligible articles are shown in Table 1 [5, 15, 16, 17, 18, 19, 20, 21, 22, 23, 24, 25, 26, 27, 28, 29, 30, 31, 32, 33, 34, 35, 36, 37, 38, 39, 40, 41, 42, 43, 44, 45, 46, 47, 48]. All studies were rated as low risk of bias. Every study reported the dose of GH used. Of the 6732 participants (2949 (44%) female) included in the studies, the overall median reported age of the participants was 49 (range 22, 82) years. Among the studies, the median number of participants was 43 (9, 1988) and the median study follow‐up period was 12 months (4, 180). Studies included both men and women, although often data were not presented for men and women separately, making it impossible to determine any sex differences. Ethnicity was only explicitly reported in 4 (13%) studies, with the majority of included patients being reported as Caucasian.

**TABLE 1:** Summary of papers included in systematic review.

First Author	Yr	Country	Study	N; Female (%)	Mean age (yrs, range)	Study Duration (months)	Adiposity	CV	IGF‐1	QoL	Other
Bengtsson [9]	1993	Sweden	n‐RCT	10;1 (10%)	34–58	7	—	—	Y	Y	Y
Wallymahmed [10]	1996	UK	Cohort	32;21 (66%)	35 (20–59)	N/A	—	—	—	Y	—
Gibney [11]	1999	UK	n‐RCT	21;9 (43%)	38 (21–48)	120	—	Y	Y	Y	—
Herschbach [12]	2001	Germany	Cohort	770;327 (42%)	24–64	6	—	—	—	Y	—
Abs [13]	2005	Sweden	Cohort	267;145 (54%)	45	12	Y	Y	Y	Y	—
Hoffmann [14]	2005	USA	RCT	135;46 (34%)	44	8	Y	—	Y	—	Y
Cenci [15]	2009	Brazil	n‐RCT	14;10 (71%)	45 (33–62)	6	—	Y	Y	—	—
Cittadini [16]	2009	Italy	RCT	28;5 (18%)	62	6	—	Y	—	—	—
Lazurova [17]	2009	Slovakia	Cohort	59; NK %	34	6	—	Y	—	—	—
Andreassen [18]	2011	Denmark	Cohort	16;8 (50%)	49	12	—	Y	Y	—	—
Gruson [19]	2011	Belgium	n‐RCT	40;19 (48%)	43	6	—	Y	—	—	—
Cittadini [20]	2013	Italy	RCT	28;5 (18%)	62	48	—	Y	—	—	—
Elbornsson [21]	2013	Sweden	n‐RCT	156;63 (40%)	51 (22–74)	180	Y	Y	Y	—	—
Hartman [22]	2013	USA	Cohort	1988;795 (40%)	46	48	—	Y	—	—	—
Barbosa [23]	2014	Sweden	Cohort	311;130 (42%)	50 (17–77)	12	—	Y	—	—	—
Brod [24]	2014	USA	Cohort	39;23 (59%)	51 (22–82)	N/A	—	—	—	Y	—
Dlesk [25]	2014	Slovakia	n‐RCT	45;24 (53%)	37	12	—	Y	—	—	—
Chikani [26]	2015	Australia	Cohort	13;12 (92%)	45	N/A	Y	Y	—	Y	—
Boschetti [27]	2016	Italy	n‐RCT	14;7 (50%)	49	12	—	Y	—	—	—
Elbornsson [28]	2017	Sweden	RCT	95;49 (52%)	53 (20–76)	84	Y	—	—	Y	—
Gonzalez [29]	2017	UK	RCT	17;7 (41%)	48 (19–74)	6	Y	Y	—	—	—
Johannsson [30]	2018	Sweden	RCT	78;30 (38%)	45 (19–65)	10	Y	—	—	—	Y
Ku [31]	2018	Europe & Korea	RCT	45;14 (31%)	N/A (20–65)	4	Y	—	Y	—	Y
Suzuki [32]	2018	Japan	n‐RCT	9;2 (22%)	44	6	Y	Y	—	—	—
van Bunderen [33]	2018	Netherlands	RCT	32;11 (34%)	47	6	Y	—	—	Y	—
Beck‐Peccoz [34]	2019	England	Cohort	1293;627 (49%)	49	N/A	—	—	Y	—	Y
Johannsson [35]	2020	Sweden	RCT	301;155 (52%)	45 (23–79)	8.5–13	Y	—	Y	—	Y
Losa [36]	2020	Sweden & Germany	Cohort	283;102 (36%)	51 (51–52)	84	—	—	—	—	Y
Lundberg [37]	2020	Sweden	Cohort	293;145 (50%)	50	120	Y	—	Y	—	Y
Otsuka [38]	2020	Japan	RCT	62;29 (47%)	53 (20–75)	13	Y	—	Y	Y	Y
Wang [39]	2020	China	n‐RCT	60;37 (62%)	49 (20–74)	12	—	Y	Y	—	—
Arosio [40]	2021	Italy	Cohort	88;37 (42%)	48.9	51.5 ± 37	—	Y	Y	—	Y
Biller [41]	2021	United States	Cohort	40;40 (100%)	33 (23–42)	N/A	—	—	—	—	Pregnancy
van Bunderen [42]	2021	Netherlands	RCT	32;11 (34%)	N/A (46–47)	6	Y	Y	—	—	—
Götherström [43]	2022	Bolivia	RCT	18;3 (17%)	53	18	—	Y	Y	Y	—

Abbreviations: CV, cardiovascular; QoL, quality of life; n‐RCT, non‐randomised controlled trial; RCT, randomised controlled trial.

**FIGURE 2:**
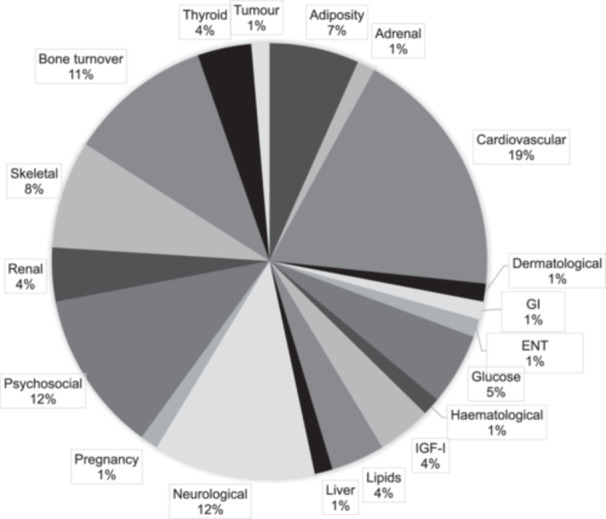
Number of different outcome measures reported according to category. Percentage shown of the 75 different outcomes reported in the included publications. ENT; ear nose and throat; GI: gastrointestinal; IGF‐I: insulin‐like growth factor 1.

The most common outcome category reported in AGHD was cardiovascular events with 21 (60%) studies reporting an outcome in this category. Table 2 demonstrates the rates of the most common outcome categories that were reported in > 10% of studies. Of the 75 different outcome measures reported overall in the included articles, 37 (49%) were associated with safety alone, 24 (32%) were associated with efficacy alone and 14 (19%) were associated with both (Table 2; Figure 2).

**TABLE 2:** Outcomes reported in > 10% studies.

Outcome category	Effectiveness	Safety	No of studies (*n* = 33)	%
**Cardiovascular**	*	*	**21**	**64**
Echocardiography	*		8	25
Blood pressure		*	8	22
**IGF‐1/BP3**	*	*	**15**	**45**
IGF‐1	*	*	13	38
IGF‐1 SDS	*	*	8	25
**Body composition**	*	*	**15**	**45**
Body mass index	*	*	8	22
Fat mass	*	*	7	19
Waist/hip circumference	*	*	6	16
Lean body mass	*	*	5	13
**Psychosocial**	*	*	**8**	**24**
QoL‐AGHDA	*	*	5	13
**Skeletal disorders**		*	**7**	**22**
Arthralgia		*	7	22
**Lipids**	*		**7**	**19**
High density lipoprotein	*		7	19
Low density lipoprotein	*		7	19
Total cholesterol	*		7	19
**Glucose homoeostasis**		*	**6**	**19**
HbA1C		*	6	19
**Neurological**		*	**4**	**13**
Headache		*	4	13

Abbreviations: BMC, bone mineral content; BMD, bone mineral density; HbA1C, glycated haemoglobin; IGF‐1, insulin‐like growth factor; IGF‐BP3, insulin‐like growth factor binding‐protein 3; QoL‐AGHDA, Quality of Life Adults with Growth Hormone Deficiency; SDS, standard deviation score; VO2 max, maximal oxygen capacity.

### 3.3 Development of the Minimum Data Set

**FIGURE 3:**
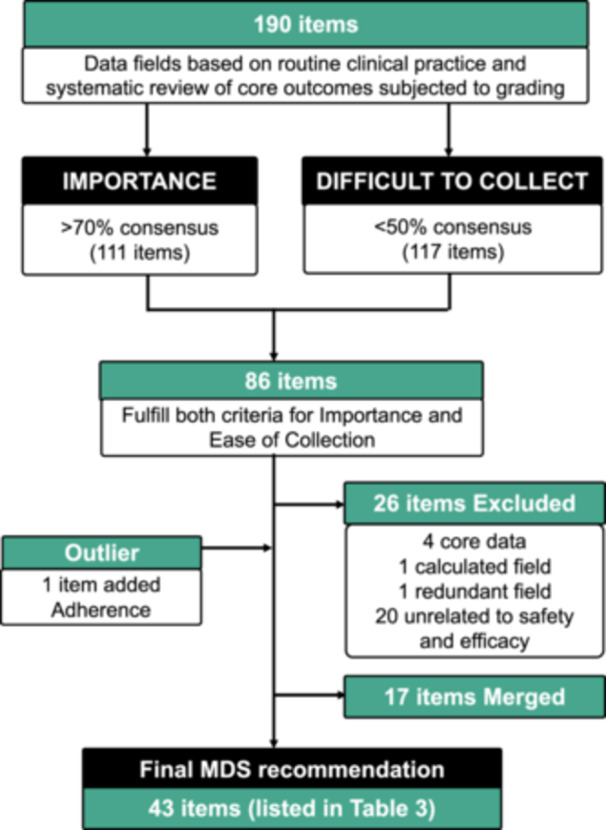
Consort diagram from initial comprehensive list to final MDS recommendation.

At baseline, the comprehensive list of data fields from routine clinical practice and those from the core outcome recommended in the systematic review generated a total of 190 items which were subjected to grading by the clinical experts. Of these, only 111 items achieved > 70% consensus as important data to collect, 44 items achieved 50%–70% consensus, and 35 items received less than 50% consensus as being important to collect. A total of 117 of the 190 items were deemed easy to collect. Combining both criteria for importance and ease of collection, 86 items qualified for the MDS. Twenty‐five of these were excluded, one being redundant (brand of GH), four being core data items (date of birth, gender, country, centre name) and 20 which were unrelated to safety or efficacy. A total of 17 items were merged into similar headings, aligning to the structure for these items in the Childhood GHD module [11] (Figure 3).

Two areas were specifically highlighted for discussion, namely adherence and quality of life. All participants unanimously agreed that information on adherence is very important to be collected, but it was deemed difficult to collect by the majority (89%), so this item did not fulfil the criteria for MDS. Participants also graded five items pertaining to HRQoL, assessing (i) fatigue, (ii) sleep quality, (iii) quantifying ability to work by number of days/week, and more objectively using the (iv) EQ. 5D or (v) AGHD questionnaires respectively. All but the last one achieved 100% consensus as being important to collect, but only one item ‘assessing fatigue’ qualified for MDS as the rest were deemed difficult to collect. These items were discussed further and examined on their individual merits with consensus reached for including adherence in the MDS and for excluding fatigue from the final MDS recommendation of 45 items, of which two are calculated fields, namely body mass index (BMI) and low‐density lipoprotein (LDL). Of the 45, 22 (50%) would only be required to be completed once, such as family history, details of pretreatment GH stimulation tests and date of start of first GH therapy to name a few (Table 3).

**TABLE 3:** Finalised GloBE‐Reg MDS for adulthood growth hormone deficiency (GHD) module.

Diagnosis	Therapy	Clinician Reported Outcomes	Adverse Events
**Past medical history**	**GH therapy**	**Anthropometry**	Since the last recorded clinical encounter in the GloBE‐Reg Registry, has the patient experienced any medication error related to the drug?
	Date of start of First GH therapy	Height (cm)	
Neoplasia or cranial tumour	Date of start of Current therapy	Weight (kg)	
Cranial or total body irradiation	GH dose	**Biochemistry**	
Chemotherapy	Adherence	IGF‐1	
Traumatic brain injury	Date of end of therapy	TSH	
Pituitary/cranial surgery	GH reinitiation after FAH	Free T4	
Other co‐morbidities/surgery	**Other medications**	HbA1c	
FHx of diabetes mellitus	Thyroid hormone		
FHx of hypertension	Glucocorticoids	Triglyceride	Since the last recorded clinical encounter in GloBE‐Reg Registry, has the patient experienced any untoward medical occurrence (sign, symptom or disease) which, by your clinical judgement, may be possibly related to the drug?
FHx of cardiovascular disease	Oestrogen	HDL	
FHx of tumour	Progesterone	Total cholesterol	
Pregnancy status	Testosterone	**Cardiovascular parameters**	
Participation in clinical trials	Oral contraception	Systolic BP	
**Biochemistry/imaging at diagnosis**		Diastolic BP	
Pituitary/brain MRI		Pulse	
IGF‐1 result			
Type of GH Stimulation Test 1			
Peak GH Test 1			
Type of GH Stimulation Test 2			
Peak GH Test 2			
TSH			
Free T4			
HbA1c			
Total cholesterol			
HDL			
Triglyceride			

Abbreviations: BP, blood pressure; FAH, final adult height; FHx, family history; GH, growth hormone; HbA1c, glycated haemoglobin; HDL, high density lipoprotein; IGF‐1, insulin like growth factor 1; LDL, low density lipoprotein; MRI, magnetic resonance imaging; TSH, thyroid stimulating hormone.

## 4 Discussion

Data about a MDS for the monitoring of safety and effectiveness of replacing GH in adults with AGHD are needed. By combining a systematic review of reported outcomes in this field with a structured and objective assessment of the level of consensus within an expert group, the current study has adopted a novel approach for informing the development of a standardised MDS that can be collected in routine clinical practice. We believe that this MDS will be sufficient to capture the crucial safety and effectiveness information on the use of GH in adults with GHD. The development of the MDS does not preclude the collection of other outcomes that may be of research interest in the future—they are designed to be the minimum for collection, but other information can be added by sites if there is a specific project that addresses other outcomes. By restricting the MDS to the absolute minimum, it is expected that the data entry burden will be decreased, resulting in more complete, high quality data entry. Lastly, having a defined and standardised MDS allows all disease registries to collect the same dataset, thus facilitating the sharing and exchange of data between several data sources and more comprehensive analyses over the longer term.

Importantly, this study has also demonstrated the discrepancies that exist between what may be considered a clinical trial dataset and the MDS which is more practicable for routine real world data collection for the monitoring of long‐term safety and efficacy of GH replacement therapy in AGHD. For instance, amongst the reported outcomes that were identified in the systematic review, the most common categories of reported outcomes were cardiovascular, IGF‐I and adiposity. However, studies measured and reported these outcomes heterogeneously and many of the reported outcome measures, for example dual X‐ray absorptiometry (DXA) for body composition would not be undertaken as part of routine clinical practice, as highlighted in the exchange with the GloBE‐Reg expert group. Triglyceride concentration collected in the fasting state and non‐fasting state have both been reported to be valuable markers of cardiometabolic risk in epidemiological studies [48, 49]. However, in clinical trials of GH therapy, lipids have only been collected in a fasting state [47, 49, 50]. It is likely that in routine clinical practice lipids will be measured in a non‐fasting state but a standardised MDS in a global registry has the potential of collecting both and generating sufficient data to demonstrate the effect of GH therapy on the lipid profile.

It is no surprise that cardiovascular outcomes are most commonly reported, given the increased likelihood of cardiovascular morbidity in AGHD, although in general, the administration of GH replacement therapy appears to positively impact the prognosis of cardiovascular diseases in these patients, with more recent sex differences noted [1, 51]. Of the studies focussing on cardiovascular outcomes, 11 were summarised in a comprehensive meta‐analysis [52]. It would not be common practice for echocardiography to be conducted on every individual with AGHD, as commented by the expert group. Surprisingly few studies measured simple blood pressure or considered rate of admission to hospital with cardiovascular disease and no studies reported cardiovascular admissions as AEs. Given that these events would be easier to identify than echocardiography or cardiovascular biomarkers, these could be considered as part of a MDS.

In studies of AGHD, the IGF‐I standard deviation score (SDS) is widely reported, whilst some preferred also the inclusion of IGFBP3, this is not readily available in every centre internationally [53, 54] and less important in adults versus children. In addition to determining the effectiveness of treatment, IGF‐I concentrations are also used to adjust GH dosage to avoid adverse reactions caused by excessive dosing of GH [55]. In adults it is considered a crucial biomarker for monitoring therapeutic safety and efficacy. In the development of the MDS, there was absolute unanimous agreement on the need to collect IGF‐I values during routine monitoring, unlike IGFBP3 which did not qualify for the MDS as the experts found the latter not very useful in AGHD. In addition, in the systematic review, some studies reported the absolute value of the biochemical parameter, some calculated SDS scores, and others only focused on whether it was within a reference range or different compared to baseline concentrations. We recommend the use of IGF‐I SDS to ensure comparisons between biochemical assays. When collecting the data for LAGH, it is also important to record the timing of the sample in relation to the last injection dose [56].

AEs can be difficult to systematically quantify in any patient group. There is a balance between asking about too many different potential AEs and missing relevant ones. There remains a need to consider the safety of any prescribed therapy, so this has been included in the MDS. In particular, one recent systematic review reported four deaths during GH therapy, although these were thought to be unrelated to GH [57].

Although health‐related quality of life (HRQoL) assessment and patient reported outcomes (PROs) were identified in the systematic review, broader challenges remain regarding the optimal definition of HRQoL and concerns that non‐condition‐specific PROs may not accurately reflect HRQoL. There are over 20 different PROs that have been used in AGHD studies [58], of which five are validated for AGHD as per the COSMIN guideline [59], specifically the Disease Impact Scale [16], Life Fulfilment Scale [16], Mental Fatigue Questionnaire [16], Quality of Life Assessment of Growth Hormone Deficiency in Adults (AGHDA) [60], and Questions on Life Satisfaction‐Hypopituitarism [18]. The most common questionnaire reported in the systematic review was the AGHDA. In some countries such as the UK, an impaired QoL as demonstrated by an AGHDA score is an essential criterion for prescribing GH in AGHD [61]. However, in the expert group, the consensus was that the guidance for collecting PROs in routine clinical practice was not sufficiently clear. The GloBE‐Reg platform has the functionality to add PROs so that they can be directly reported and viewed by patients and there is a need to explore how these tools can now be used in routine clinical practice at a global level.

In conclusion, the systematic review of outcomes that have been reported in studies of GH replacement in AGHD, in combination with a structured consultation with an expert group in GloBE‐Reg has allowed the development of a MDS that we believe should be collected in all AGHD patients who are receiving GH. This MDS would allow the reporting of commonly reported outcomes, or core outcomes, that were identified in the systematic review by all interested sites around the world. The identification of this MDS will improve the comparability and consistency of results across different studies, enhancing the quality of evidence and facilitating evidence‐based decision‐making in the field of GH treatment for AGHD.

## Disclosure

S.C.C has received consulting honoraria from Pfizer and was PI of research funding from Pfizer to the Royal Hospital for Children Glasgow. B.M.K.B is the PI of research funding from Ascendis to Massachusetts General Hospital and occasional consulting honoraria from Ascendis (unrelated to the research) and Novo Nordisk. P.E.C. is a recipient of grant funding from Novo Nordisk, Copenhagen, Denmark, and is a Consultant & Senior Medical Advisor to Lumos Pharma, Austin, Texas. M.F. has received research funding to the University as PI from Ascendis and has received occasional consulting fees from Novo Nordisk for unrelated projects. K.K.Y.H. has received fees from Novo Nordisk. J.O.L.J. has received fees from Novo Nordisk and Pfizer. X.L. is advisor and PI for GenSci, Novo Nordisk, Takeda, Ipsen, Amoytop. B.S.M. has received consultant fees from Abbvie, Ascendis Pharma, BioMarin, Bristol Myers Squibb, EMD Serono, Novo Nordisk, Orchard Therapeutics, Pfizer and Tolmar as well as grants from Alexion, Abbvie, Adrenas Therapeutics, Aeterna Zentaris, Lumos Pharma, Lysogene, Novo Nordisk, OPKO Health, Pfizer and Spruce Biosciences. S.N. has received research grants from Pfizer, consultancy and/or speakers from Novo Nordisk and Crinetics. K.S. has received research funding to the University as PI from Novo Nordisk and Ascendis. C.J.S. served as occasional paid consultant/speaker for Sandoz‐Hexal, NovoNordisk, Pfizer, ConsilientHealth, Pharmanovia, Recordati, Debiopharm, and Crinetics, Y.T. has received lecture and consultancy fees and from Novo Nordisk. G.J. has received fees and grant from Ascendis Pharma, Astra Zeneca, Novo Nordisk, Pfizer, Sandoz and Shire. K.C.J.Y. has received research grants to his institution form Ascendis, Corcept, Chiesi and Sparrow, and has received honoraria from Novo Nordisk, Ascendis, Chiesi, Recordati, Xeris, Crinetics, Camurus and Neurocrine. ARH has received consultancy fee from Ascendis. G.J. has received support from Ascendis Pharma, Astra Zeneca, Novo Nordisk, Pfizer, Sandoz and Shire. S.F.A. has received research grants from GenSci, Novo Nordisk and Pfizer for the GloBE‐Reg project. A.L.H., R.T., X.T., M.A., D.A., J.B., M.C., J.G., L.S. and D.V. have nothing to disclose.

## Data Availability

Some or all datasets generated during and/or analysed during the current study are not publicly available but are available from the corresponding author on reasonable request.
